# Women's Work in War

**Published:** 1910-12-10

**Authors:** 


### The Hospital

A Journal of

The Medical Sciences and Hospital Administration.

New Series. No. 200, Vol. VIII. [No. 1272, Vol. XLIX.] SATURDAY, DECEMBER 10, 1910.

### WOMEN'S WORK IN WAR.

Hitherto the main service that womanhood has rendered in time of war has been of a moral character. The brunt of silent endurance and resigned suffering has always been borne by the wives, mothers, and daughters of the men in the fighting lines. The hypothesis that the female sex can, and ought to, take a more active share in bearing the apparent burdens of war is one to which no unprejudiced mind is likely to agree. No one wishes to breed a race of Amazons, with an Antiope in each county. But it is quite another matter when we come to the question, "Are there other ways in which women may help in a national emergency of such magnitude as war?"

The obvious answer is that they may do excellent service in the nursing department. The history of the late Florence Nightingale and of the many women who have, since the Crimean campaign, emulated her example shows clearly how wide is the scope for true womanly service in this direction. But it is worth while to consider whether women's work in time of war stops short at nursing in home or hospital, or whether it is not possible to extend its scope so as to include departments in which service has hitherto been considered as essentially "men's work." For instance, the Army Medical Corps absorbs a large quota of able-bodied men whose services would be of immense importance in the fighting line. In present circumstances these men are non-combatants. It is at least arguable that if they were replaced by a thoroughly efficient and well-trained women corps, the Army as a whole would gain by the innovation.

That is the premiss which the ladies responsible for the establishment of the Women's Convoy Corps have considered, and the movement which has led to the inception of the corps is an endeavour to show on what lines such field service may be accomplished by women. The objects of the Corps are to act as relieving units, adjuncts to the Territorial Army Medical Corps. There is no ambition to move about with skirmishing columns, and pick up the wounded on the battlefield; all that is provided for by the Army Medical Corps and accessory units working in co-operation with it. But the Convoy Corps stands intermediate between this latter group and the Voluntary Aid Detachments, which, under the Red Cross Society's scheme, will have to deal with the wounded in the base hospitals. It furnishes recruits and units to these detachments, but, more than that, it is itself able and willing to act as a collecting station—not in the old sense of the term—and to undertake the transport of the sick and wounded. Its efficiency, both from a physical and regimental point of view, is, we understand, already high, and in course of time will undoubtedly increase.

Without entering into the merits of the particular form which this new movement has taken in the creation of such a special entity as the Convoy Corps—a form which is obviously open to criticism in some directions—it may be postulated that the influence of the movement is bound to be beneficial to the nation at large. For it is, after all, an admission that these women who have joined the Corps realise the fact that true citizenship entails duties and obligations which should be cheerfully fulfilled even at the cost of some self-sacrifice. That these obligations are for women no less than for men we now generally acknowledge: even the most rabid "anti-feminist" will agree with Mary Astell that moral civic education is as much a necessity for one sex as for the other. It is a great gain to have such an admission, and it is significant that the movement, to some extent at least, synchronises with the public realisation in other quarters of important national responsibilities. All this makes the future outlook brighter for those of us who hold that it is an essential duty of the State to train its citizens to defend their homes. The acute fear of militarism which exists in some quarters will doubtless prejudice some well-meaning people against the movement, just as it promotes the vigorous opposition to compulsory military-training of the nation's youth, though there can be little doubt that such training will, from its very nature, be one of the best guarantees of peace and one of the surest safeguards against aggressive Jingoism on a dangerous scale. The influence which these women are likely to have is immense. As mothers and wives they will to a large extent be responsible for the mental attitude which their husbands and sons will take up in regard to the question of defensive military training. Their acquaintance, derived from training in the field, with the results of war in one aspect at least, will enable them to realise fully the supreme importance of thoroughness and preparation. It will make them feel that prophylaxis is as necessary for the nation as for the individual, and that there can be no question of waiting for an emergency to arise in order to prepare for and cope with it. Once these important facts have been thoroughly grasped their gradual percolation through the mass of the community upon which the influence of members of the Corps reacts is only a matter of time.

Moreover, the importance of the training that these women receive does not lie merely in its value in time of national stress. It lies even more in the fact that such training tends to promote physical efficiency, both individual and national, in all directions. The principles of hygiene, domestic economy, first aid, sanitation, preventive medicine, and, above all, discipline, self-reliance, and solidarity which it inculcates are of enormous value in times of peace. When it is remembered that much of this training is unbroken ground for a large percentage of otherwise well-educated women, it is difficult to overestimate its true worth or to deny its educative influence upon the community. There are great sociological problems confronting us in the decade to come, and it should be the interest of every citizen to do his or her utmost towards furthering their solution. That can only be done by education, by training, by discipline, and, be it added, by an amount of personal service and self-sacrifice which it needs some hardihood to give. Is it too much to hope that the establishment of such volunteer organisations as the Women's Sick and Wounded Convoy Corps will assist in impressing upon the nation the fact that true citizenship means personal service?

